# A New Failure Theory and Importance Measurement Analysis for Multidirectional Fiber-Reinforced Composite Laminates with Holes

**DOI:** 10.3390/ma15062227

**Published:** 2022-03-17

**Authors:** Shu Li, Fei Han

**Affiliations:** Department of Mechanical and Electrical Engineering, North China University of Technology, Beijing 100144, China; hanfei@ncut.edu.cn

**Keywords:** fiber-reinforced composite laminate, hole, failure, notched strength, importance measurement, uncertainty

## Abstract

In this paper, a failure theory for the multidirectional fiber-reinforced composite laminate with a circular hole is developed. In this theory, the finite fracture mechanics method is combined with the improved Puck’s failure theory including the in situ strength effect. It can predict the notched strength by only basic material properties of unidirectional laminas, geometries and stacking sequence of the laminate. In advance mechanical properties of the laminate are unnecessary. The notched laminates with different material types and stacking sequences are taken as examples to verify this failure theory, and predicted results are in good agreement with experiments. Based on the developed failure theory, importance measurement of uncertain material properties to the notched strength is analysed. Results show that notched strength increases with increasing longitudinal tensile strength and in-plane shear modulus for the laminate with an arbitrary hole diameter. However, it decreases with increasing transverse modulus.

## 1. Introduction

The composite laminate with open holes is a common structure for connection in the aerospace industry, and the hole may weaken structural integrity and then cause stress concentration. Therefore, a method which can predict failure strength accurately and fast is necessary for structural design. Although various non-linear finite element (FE) approaches coupling with progressive failure models are accurate enough [1,2], these methods are still not acceptable for preliminary sizing because they are time consuming [3].

The most widely used design method suitable for preliminary sizing of notched composite laminates is the average stress or point stress criterion developed by Whitney and Nuismer [4]. It is assumed that failure takes place when the average stress over a distance or stress at a point with a given distance from the hole boundary (the `characteristic distance’) reaches the unnotched strength of the laminate. The characteristic distance in both average stress and point stress criteria is obtained from a test of a notched laminate. Research has shown that the characteristic distance is not an inherent material property, it is also related to stacking sequence and geometry [5]. Thus, many expensive experiments should be carried out to identify the characteristic distance of the notched laminate with different materials, stacking sequences and geometries. In order to avoid determining the characteristic distance by large experimental programmes, the finite fracture mechanics (FFMs) model is developed from the concept of finite fracture mechanics originally proposed by Leguillon [6]. FFMs model assumes that failure occurs when a stress-based criterion and an energy-based criterion are fulfilled simultaneously [3,7]: average stress criterion, energy for crack propagating a finite distance reaching the fracture toughness. There are also some variations of FFMs model proposed by Felger et al. [8] and Reinoso et al. [9].

Among FFMs models, mechanical information of the unnotched laminate should be known in advance. The unnotched tensile strength is one of prior and important physical quantities, and can be obtained by experiment [3] or failure criterion [10]. However, experiments are always expensive and time consuming. In contrast, high-precision failure criteria are less time consuming, i.e., fast. As for failure criteria, Puck’s theory considering fiber failure and inter-fiber failure is proved effective on unnotched strength analysis of multidirectional composite laminates in world wide failure exercise-I, II [11,12]. However, predicted initial strength from Puck’s theory is smaller than experimental data. To overcome this, Dong et al. proposed the improved Puck’s theory with the in situ strength effect which considers the influence of both the lamina itself and its neighbouring laminae [13]. Li and Ma introduced the improved Puck’s theory with the in situ strength effect into strength analysis of multidirectional intact composite laminate with uncertain material properties [14]. In this paper, a new failure theory for notched fiber-reinforced composite laminates will be developed, by combining the FFMs model with the improved Puck’s theory with in situ strength effect. In the developed failure theory, only basic material properties of unidirectional laminas, geometries and stacking sequence of the laminate are needed.

Besides accurate and fast strength prediction models, uncertainty of the fiber-reinforced composite laminate is also an important problem to discuss. The uncertainties result from various forms of defects [15], manufacturing process [16] and experimental measurement. All these uncertainties cause uncertain material properties [17,18,19]. Material properties are always as input variables in the strength prediction model, and uncertain material properties may cause the predicted failure strength deviating from the average value. Therefore, importance measurement analysis of the material properties is significant. Many related researches have been studied, such as in-plane failure probability of composite laminates with random strength parameters of unidirectional lamina [20], uncertainties of unidirectional [17] and multidirectional [10] composite strength, the influence of random geometry on notched tensile strength [21] and so on. However, to the authors’ knowledge, the importance measurement of unidirectional laminas’ material properties to the notched strength of a fibre-reinforced composite laminate is yet to be investigated.

The main goals of this paper are to develop an accurate and fast (i.e., less time consuming) failure model for notched composite laminate, and to analyse sensitivity of the uncertain materials on notched strength. The paper is organized as follows: In Section 2, we will present the developed failure model derived from FFMs method combined with the improved Puck’s failure theory including the in situ strength effect. The description of the four factors (stress distribution, stress intensity factors, unnotched strength, mode I fracture toughness) in the model is shown in detail. In Section 3, the Sobol’s global sensitivity indices are introduced to indicate the sensitivity of the uncertain materials on notched strength reliability of the composite laminate. Section 4 reports the predicted notched strength, and sensitivity analysis of material properties to notched strength. Finally, Section 5 concludes the paper with major work and highlights.

## 2. The Failure Model of the Notched Laminate

**Figure 1:**
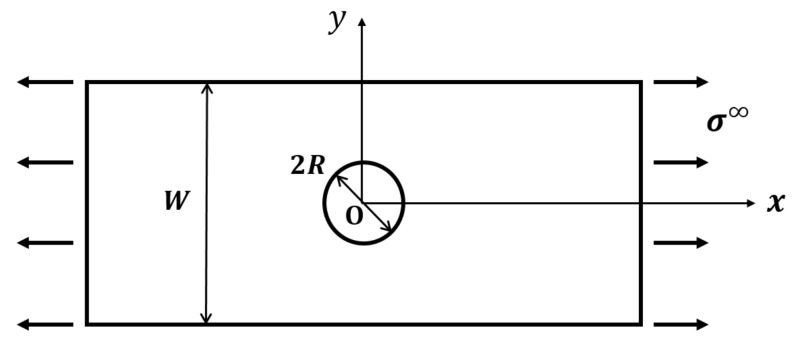
The model of a notched laminate under tensile loading.

A composite laminate with a central circular hole is shown in Figure 1, and the laminate is under tensile loading in the *x*-direction.

The laminate is with width *W*, and the hole is with radius *R*. It is assumed that the macro-crack leading to final failure is along the *y*-direction. The coupled FFMs criterion considering both the average stress model and energy balance during crack propagation is used in this paper, and the detail of this criterion is [3,6,7]

(1) $$\left\{\begin{array}{cc} \frac{1}{l}\int_{R}^{R+l}\sigma_{xx}\left(0,y\right)dy & =X_{T}^{L}\\ \frac{1}{l}\int_{R}^{R+l}K_{I}^{2}\left(a\right)da & =K_{\mathrm{IC}}^{2} \end{array}\right.,$$

where $X_{T}^{L}$ and $K_{\mathrm{IC}}$ represent the unnotched strength and mode I fracture toughness of the laminate, respectively. *l* represents the crack extension at failure. $K_{I}$ represents the stress intensity factor of the crack emanating from the hole, and $\sigma_{xx}\left(0,y\right)$ represents the stress distribution along the *y*-axis. Instead of experiments, the theoretical expressions of the four physical quantities ( $\sigma_{xx}$ , $K_{I}$ , $X_{T}^{L}$ , $K_{\mathrm{IC}}$ ) obtained by the basic properties of the lamina and stacking sequence of the laminate will be used in this paper, which makes the new failure model more general. After getting the expressions of these four physical quantities, *l* and the notched strength will be obtained from (1).

### 2.1. Description of the Stress Distribution $\sigma_{xx}$

The stress distribution along the *y*-direction, $\sigma_{xx}\left(0,y\right)$ , is expressed as [22]

(2) $$\sigma_{xx}\left(0,y\right)=\sigma^{\infty }\cdot \frac{R_{k}}{2}\cdot \left[2+\xi^{2}+3\xi^{4}-\left(K_{T}^{\infty }-3\right)\left(5\xi^{6}-7\xi^{8}\right)\right],\;\;\;\xi =\frac{R}{y}$$

where $\sigma^{\infty }$ is the remote stress. $K_{T}^{\infty }$ denotes the stress concentration factor (SCF) at the hole edge of an infinite laminate, and is defined as [22]

(3) $$K_{T}^{\infty }=1+\sqrt{\frac{2}{A_{22}}\left(\sqrt{A_{11}A_{22}}-A_{12}+\frac{A_{11}A_{22}-A_{12}^{2}}{2A_{66}}\right)},$$

where $A_{ij}\left(i,j=1,2,6\right)$ are the effective laminate stiffness. $R_{k}$ denotes the finite width correction factor, which is the ratio of SCF of a finite-width laminate ( $K_{T}$ ) to SCF of an infinite laminate ( $K_{T}^{\infty }$ ). It is defined as [22]

(4) $$R_{k}=\frac{K_{T}}{K_{T}^{\infty }}=\frac{1}{\frac{3\left(1-\frac{2R}{W}\right)}{2+{\left(1-\frac{2R}{W}\right)}^{3}}+\frac{1}{2}{\left(\frac{2R}{W}M\right)}^{6}\cdot \left(K_{T}^{\infty }-3\right)\cdot \left[1-{\left(\frac{2R}{W}M\right)}^{2}\right]},$$

where $M^{2}=\frac{\sqrt{1-8\left[\frac{3\left(1-2R/W\right)}{2+{\left(1-2R/W\right)}^{3}}-1\right]}-1}{2{\left(2R/W\right)}^{2}}$ .

### 2.2. Description of the Stress Intensity Factor $K_{I}$

$K_{I}$ is the stress intensity factor of the symmetric cracks emanating from the hole edge of an anisotropic plate, and is expressed as [23]

(5) $$K_{I}\left(a\right)=\sigma^{\infty }\cdot Y\left(\rho \right)\cdot F\left(\frac{a}{W}\right)\cdot \sqrt{\pi a},$$

where *Y* is the correction factor for an anisotropic laminate, *F* is the shape function, *a* is the crack size.

*Y* is defined as [23]

(6) $$Y\left(\rho \right)=1+0.1\left(\rho -1\right)-0.016{\left(\rho -1\right)}^{2}+0.002{\left(\rho -1\right)}^{3},$$

where $\rho =\frac{\sqrt{E_{x}E_{y}}}{2G_{xy}}-\sqrt{\nu_{xy}\nu_{yx}}$ . Note that (6) and ρ are originally derived for orthotropic materials. However, they can be also used for general anisotropic laminates if the coupling effect is ignored. Furthermore, for isotropic laminates, Y=1 and ρ=1 .

For a notched laminate with two symmetric cracks at the hole edge, *F* is defined as [24]

(7) $$F\left(\frac{a}{W}\right)=F_{h}F_{w},$$

where

(8) $$\left\{\begin{array}{cc} f_{n} & =1+0.358\lambda +1.425\lambda^{2}-1.578\lambda^{3}+2.156\lambda^{4},\;\;\;\lambda =\frac{R}{a}\\ F_{h} & =\sqrt{1-\frac{R}{a}}f_{n}\\ F_{w} & =\sqrt{\sec\left(\frac{\pi R}{W}\right)\sec\left(\frac{\pi a}{W}\right)} \end{array}\right..$$

### 2.3. Description of the Unnotched Strength of the Laminate $X_{T}^{L}$

The unnotched strength of the laminate can be obtained by the progressive damage analysis method instead of expensive experiments, which contains two main parts: (I) constitutive relations for strains and stresses in the laminate and each lamina; (II) a failure criterion with the initial failure criteria and the final failure criteria. In this paper, the linear classical laminate theory (CLT) is used to get strains and stresses in the laminate and each lamina during the progressive damage process. The improved Puck’s failure theory including the in situ strength effect is used for failure analysis [13]. It has been verified that the improved Puck’s failure theory is suitable for laminated composites [10,13]. This failure theory contains the fiber failures and inter-fiber failures, and a brief review of theses two failure types will be shown below.

As to fiber failures, there are two different failure modes defined as [13]

(9) $$\left\{\begin{array}{c} \frac{\sigma_{1}}{X_{t}}-\nu_{12}\frac{\sigma_{2}}{X_{t}}+\nu_{f12}\frac{E_{1}}{E_{f1}}\cdot m_{\sigma f}\cdot \frac{\sigma_{2}}{X_{t}}=1,\;\;\;\sigma_{1}>0\\ \left|\frac{\sigma_{1}}{X_{c}}-\nu_{12}\frac{\sigma_{2}}{X_{c}}+\nu_{f12}\frac{E_{1}}{E_{f1}}\cdot m_{\sigma f}\cdot \frac{\sigma_{2}}{X_{c}}\right|+{\left(10\gamma_{12}\right)}^{2}=1,\;\;\;\sigma_{1}<0 \end{array}\right.,$$

where $\left\{X_{t},X_{c}\right\}$ are the tensile and compressive strengths of the lamina in the fiber direction, $\left\{E_{1},E_{f1}\right\}$ are the longitudinal moduli of the lamina and the fiber, $\left\{\nu_{12},\nu_{f12}\right\}$ are the Poisson ratios of the lamina and the fiber. $\left\{\sigma_{1},\sigma_{2}\right\}$ are the longitudinal and transverse normal stresses of the lamina, $\left\{\gamma_{12}\right\}$ is the in-plane shear strain of the lamina. $m_{\sigma f}$ is the ‘stress magnification effect’ because of the mismatch between the moduli of fibers and matrix. It is noted that these two fiber failure modes denote final failure.

The inter-fiber failures include three different modes defined by [13]

(10) $$\left\{\begin{array}{cc} \; & \mathrm{Mode}\;A:\;\;\;\;\;\sqrt{{\left(\frac{\tau_{12}}{S_{12}^{I}}\right)}^{2}+{\left(1-p_{⊥||}^{\left(+\right)}\frac{Y_{t}^{I}}{S_{12}^{I}}\right)}^{2}{\left(\frac{\sigma_{2}}{Y_{t}^{I}}\right)}^{2}}+p_{⊥||}^{\left(+\right)}\frac{\sigma_{2}}{S_{12}^{I}}=1-\left|\frac{\sigma_{1}}{\sigma_{1D}}\right|,\;\sigma_{2}⩾0\\ \; & \mathrm{Mode}\;B:\;\;\;\;\;\frac{\sqrt{\tau_{12}^{2}+{\left(p_{⊥||}^{\left(-\right)}\sigma_{2}\right)}^{2}}+p_{⊥||}^{\left(-\right)}\sigma_{2}}{S_{12}^{I}}=1-\left|\frac{\sigma_{1}}{\sigma_{1D}}\right|,\;\sigma_{2}<0\;\mathrm{and}\;0⩽\left|\frac{\sigma_{2}}{\tau_{12}}\right|⩽\frac{R_{⊥⊥}^{A}}{\tau_{12c}}\\ \; & \mathrm{Mode}\;C:\;\;\;\;\;\left[\;{\left(\;\frac{\tau_{12}}{2\;\left(1\;+\;p_{⊥⊥}^{\;\left(\;-\;\right)\;}\;\right)S_{12}^{I}}\;\right)}^{2}\;+\;{\left(\frac{\sigma_{2}}{Y_{c}}\right)}^{2}\;\right]\;\frac{Y_{c}}{\;-\sigma_{2}}\;=\;1\;-\;\left|\frac{\sigma_{1}}{\sigma_{1D}}\right|,\;\sigma_{2}\;<\;0\;\mathrm{and}\;0\;⩽\;\left|\frac{\tau_{12}}{\sigma_{2}}\right|\;⩽\;\frac{\tau_{12c}}{R_{⊥⊥}^{A}} \end{array}\right.,$$

where $\left\{Y_{t}^{I},S_{12}^{I}\right\}$ are the transverse tensile and in-plane shear strengths of the lamina embedded in the laminate with in situ effect, $Y_{c}$ is the transverse compressive strength of the isolated lamina, $\frac{\sigma_{1}}{\sigma_{1D}}$ denotes the degradation of the fracture resistance because of single fiber failure, $\left\{p_{⊥||}^{\left(+\right)},p_{⊥||}^{\left(-\right)},p_{⊥⊥}^{\left(-\right)}\right\}$ are constants related to the material of the laminate. The expressions of the parameters mentioned above have been shown in detail in the work of Wang [13]. If the initial or intermediate failure occurs based on the inter-fiber failure criteria, the stiffness $\left\{E_{2},G_{12},\mu_{12}\right\}$ is gradually reduced by a degradation factor η , i.e.,

(11) $$\left\{\begin{array}{c} \mathrm{Mode}\;A:\;\;\;\;\;\;\;\;\;\;\;\;\;\;\;\;\;\;\;\;\;\left\{E_{2},G_{12},\nu_{12}\right\}\to \left\{\eta E_{2},\eta G_{12},\eta \nu_{12}\right\}\\ \mathrm{Mode}\;B\;\mathrm{and}\;\mathrm{Mode}\;C:\;\;\;\left\{E_{2},G_{12},\nu_{12}\right\}\to \left\{E_{2},\eta G_{12},\nu_{12}\right\} \end{array}\right.,$$

where $\eta =1/f_{E}\left(f_{E}⩾1\right)$ and $f_{E}$ denotes the left hand terms of (10). Furthermore, the final failure will occur when the angle of the fracture plane in Mode C ( $\theta_{fp}^{C}$ ) satisfies $\tan\theta_{fp}^{C}⩾3\mu$ , where μ denotes the coefficient of friction.

### 2.4. Description of the Mode I Fracture Toughness of the Laminate $K_{\mathrm{IC}}$

In the work of Camanho et al. [3], it is noted that $K_{\mathrm{IC}}$ obtained from Equation (1) combined with experimental data of $\sigma^{\infty }$ and $X_{T}^{L}$ is close to that obtained from linear-elastic fracture mechanics. It means that $K_{\mathrm{IC}}$ in the FFMs model can be obtained by the fracture toughness of the $0^{{}^{\circ}}$ lamina without producing significant errors [3], and the corresponding relationship between them is as follows.

For an anisotropic composite laminate, Ω denotes the ratio of the failure stress of the sublayer (labeled by a superscript (*k*)) to $0^{{}^{\circ}}$ layer [25], i.e.,

(12) $$\Omega_{0}^{\left(k\right)}=\frac{{\bar{\sigma }}^{\left(k\right)}}{{\bar{\sigma }}^{0}},$$

where ${\bar{\sigma }}^{0}$ and ${\bar{\sigma }}^{\left(k\right)}$ are the remote stresses for $0^{{}^{\circ}}$ layer and the sublayer labeled *k* when final failure occurs, and these two parameters can be obtained by the improved Puck’s failure theory considering stress redistribution caused by matrix cracks mentioned in Section 2.3. Based on Equation (5), the failure stresses of the sublayer and $0^{{}^{\circ}}$ layer are expressed as

(13) $$\left\{\begin{array}{cc} {\bar{\sigma }}^{\left(k\right)} & =\frac{K_{\mathrm{IC}}^{\left(k\right)}}{Y^{\left(k\right)}\cdot F\left(\frac{a}{W}\right)\cdot \sqrt{\pi a}}\\ {\bar{\sigma }}^{0} & =\frac{K_{\mathrm{IC}}^{0}}{Y^{0}\cdot F\left(\frac{a}{W}\right)\cdot \sqrt{\pi a}} \end{array}\right..$$

Substituting Equation (13) to Equation (12), $K_{\mathrm{IC}}^{\left(k\right)}$ is obtained as

(14) $$K_{\mathrm{IC}}^{\left(k\right)}=\frac{Y^{\left(k\right)}}{Y^{0}}\cdot \Omega_{0}^{\left(k\right)}\cdot K_{\mathrm{IC}}^{0}.$$

According to the relationship between the fracture toughness and the energy release rate, the energy release rate of the sublayer is [26]

(15) $$G_{\mathrm{IC}}^{\left(k\right)}=\frac{{\left[K_{\mathrm{IC}}^{\left(k\right)}\right]}^{2}}{E_{eq}^{\left(k\right)}},$$

where the effective modulus $E_{eq}^{\left(k\right)}$ of the sublayer is

(16) $$E_{eq}^{\left(k\right)}=\frac{\sqrt{2E_{y}^{\left(k\right)}E_{x}^{\left(k\right)}}}{\sqrt{\sqrt{\frac{E_{y}^{\left(k\right)}}{E_{x}^{\left(k\right)}}}+\frac{E_{y}^{\left(k\right)}}{2G_{xy}^{\left(k\right)}}-\nu_{xy}^{\left(k\right)}}}.$$

The energy release rate of the whole composite laminate is

(17) $$G_{\mathrm{IC}}^{L}=\frac{\sum_{k=1}^{N}G_{\mathrm{IC}}^{\left(k\right)}\cdot t^{\left(k\right)}}{t^{L}},$$

where *N* is the number of plies, $t^{L}$ is the total thickness of the laminate and $t^{\left(k\right)}$ is the thickness of the sublayer.

Based on (15) and (17), the mode I fracture toughness of the laminate $K_{\mathrm{IC}}$ is

(18) $$K_{\mathrm{IC}}=\sqrt{G_{\mathrm{IC}}^{L}\cdot E_{eq}^{L}},$$

where $E_{eq}^{L}$ is the effective modulus of the laminate.

Until now, $\sigma_{xx}\left(0,y\right)$ , $K_{I}$ , $X_{T}^{L}$ and $K_{\mathrm{IC}}$ in Equation (1) have been obtained by the basic properties of the lamina and stacking sequence of the laminate. Substituting (2), (5), (18) and the unnotched strength $X_{T}^{L}$ to (1), the crack extension at failure *l* is obtained from the following equation:

(19) $$\frac{4\pi l\int_{R}^{R+l}{\left(F_{h}F_{w}\right)}^{2}ada}{R_{k}^{2}{\left\{\int_{R}^{R+l}\left[2+\xi^{2}+3\xi^{4}-\left(K_{T}^{\infty }-3\right)\left(5\xi^{6}-7\xi^{8}\right)\right]dy\right\}}^{2}}=\frac{1}{Y^{2}}{\left(\frac{K_{\mathrm{IC}}}{X_{T}^{L}}\right)}^{2}.$$

From the analysis of Equation (19), the integral in the numerator of (19) can be solved numerically by Simpson’s rule and that in the denominator can be solved analytically. Once *l* is determined, the remote stress at failure or failure strength of the laminate can be obtained by one of Equation (1).

## 3. Sensitivity Analysis of Uncertainties

The sensitivity analysis (SA) is of great importance in structural safety designing considering uncertainties. SA can be classified into two types: local sensitivity analysis and global sensitivity analysis. Among these two methods, global sensitivity analysis is the most widely used because it can measure the influence of uncertain input variables on output variables in the whole distribution area. In this paper, the sensitivity of the uncertain materials on notched strength reliability of the composite laminate is assessed by Sobol’s global sensitivity indices [27,28]. A brief introduction of this method will be shown next.

There are some input variables of which the total number is *m*. The input variables can be expressed as

(20) $$X=\left[X_{1},X_{2},\cdots ,X_{m}\right],$$

and the response function is

(21) $$Y=f\left(X\right)=f\left(X_{1},X_{2},\cdots ,X_{m}\right),\;\;f:R^{m}\to R.$$

Sobol proposed the variance-based importance measurement indices using ANOVA decomposition [29]. It is noted that ANOVA decomposition is unique when the components of *X* are independent of each other, and the components are orthogonal to each other. The ANOVA-representation of response function is

(22) $$f\left(X_{1},X_{2},\cdots ,X_{m}\right)=f_{0}+\sum_{i}f_{i}\left(X_{i}\right)+\sum_{i}\sum_{j>i}f_{ij}\left(X_{i},X_{j}\right)+\cdots +f_{12\cdots m},$$

where the total number of summands is $2^{m}$ . Furthermore, the expression of constant term is

(23) $$f_{0}=E\left(Y\right)=\int f\left(X\right)\prod_{i=1}^{m}\left[g_{X_{i}}\left(x_{i}\right)d_{x_{i}}\right],$$

where $g_{X_{i}}\left(x_{i}\right)$ represents probability density function of $X_{i}$ . The first order term is obtained as the remain part when $f_{0}$ is subtracted from the mathematical expectation of the response function for other variables except $X_{i}$ , i.e.,

(24) $$f_{i}\left(X_{i}\right)=E\left(Y|X_{i}\right)-E\left(Y\right)=\int f\left(X\right)\prod_{j\neq i}^{m}\left[g_{X_{j}}\left(x_{j}\right)d_{x_{j}}\right]-f_{0}.$$

The second order term is

(25) $$f_{ij}\left(X_{i},X_{j}\right)=E\left(Y|X_{i},X_{j}\right)-f_{i}-f_{j}-E\left(Y\right)=\int f\left(X\right)\prod_{k\neq i,j}^{m}\left[g_{X_{k}}\left(x_{k}\right)d_{x_{k}}\right]-f_{i}-f_{j}-f_{0}.$$

and higher order terms can also be obtained by analogy method.

According to orthogonality of decomposition terms, the corresponding variance decomposition of (22) is

(26) $$V\left(Y\right)=\sum_{i}V_{i}+\sum_{i}\sum_{j>i}V_{ij}+\cdots +V_{12\cdots m},$$

where

(27) $$V_{i}=V\left(f_{i}\left(X_{i}\right)\right)=V\left(E\left(Y|X_{i}\right)\right),$$

(28) $$V_{ij}=V\left(f_{ij}\left(X_{i},X_{j}\right)\right)=V\left(E\left(Y|X_{i},X_{j}\right)\right)-V\left(E\left(Y|X_{i}\right)\right)-V\left(E\left(Y|X_{j}\right)\right).$$

and so on for higher orders. The total variance (unconditional variance) is obtained as

(29) $$V\left(Y\right)=\int f^{2}\left(X\right)g_{X}\left(x\right)dx-f_{0}^{2},$$

where $g_{X}\left(x\right)$ is the joint probability density function of *X*.

The global sensitivity index based on variance is defined as the ratio of right side items in (26) to total variance. Therefore, the total effect index of $X_{i}$ is

(30) $$S_{Ti}=S_{i}+\sum_{j>i}S_{ij}+\cdots +S_{12\cdots m}=\frac{V_{i}}{V}+\sum_{j>i}\frac{V_{ij}}{V}+\cdots +\frac{V_{12\cdots m}}{V},$$

and it means the contribution of $X_{i}$ itself and interaction with other variables to output response’s variance.

## 4. Results and Discussion

### 4.1. The Failure Strength of the Notched Laminate

In this section, we apply the developed failure model in Section 2 to multidirectional fiber-reinforced composite laminates. Three kinds of materials (IM7-8552, AS4/3502, Hexcel F593 epoxy system) are used to validate the new failure model for composite laminates with arbitrary stacking sequences. Furthermore, the material properties of unidirectional laminas are listed in Table 1.

**Figure 2:**
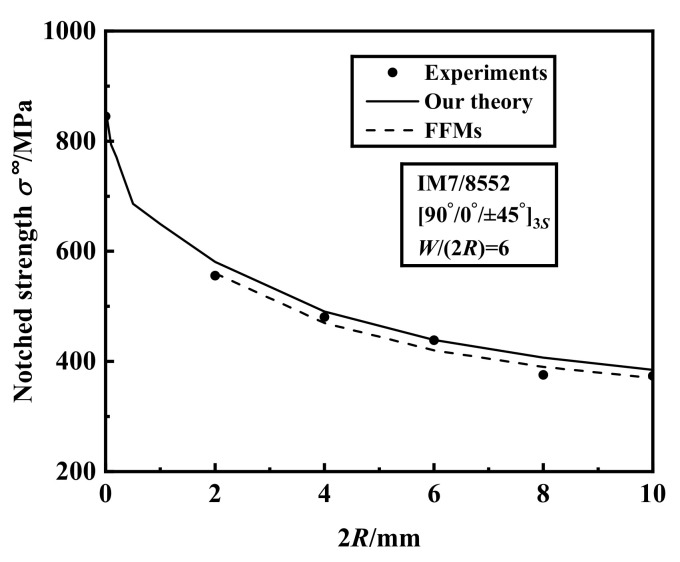
The uniaxial tensile strength of ${\left[90^{{}^{\circ}}/0^{{}^{\circ}}/\pm 45^{{}^{\circ}}\right]}_{3s}$ IM7-8552 notched laminates with varying hole diameters.

The quasi-isotropic ${\left[90^{{}^{\circ}}/0^{{}^{\circ}}/\pm 45^{{}^{\circ}}\right]}_{3s}$ notched laminates with IM7-8552 are chosen as the first batch of examples. All the laminates are with a constant width-to-diameter ratio ( $\frac{W}{2R}$ ) which is equal to 6, and with five varying hole diameters: 2R=2mm , 4mm , 6mm , 8mm , 10mm . Figure 2 presents a comparison of the experimental results from Ref. [3] and the predictions of our theory for the laminate under the uniaxial tension in the *x*-direction. The figure shows that the predictions of our theory for notched strength agree well with experiments, and the maximum error is 8.25%. It is noted that FFMs criterion is also accurate. However, FFMs method used in Ref. [3] requires the static strength of the laminate in advance imposing its limited application, whereas our predictions are only based on the basic properties of the lamina and stacking sequence of the laminate.

**Figure 3:**
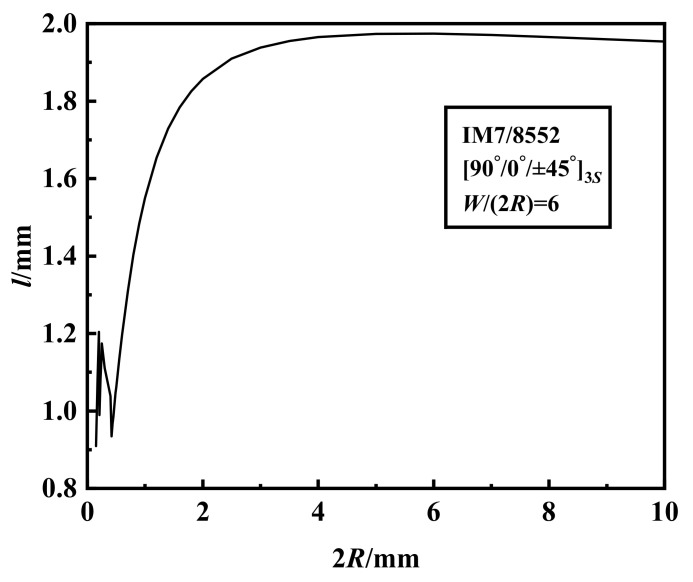
The crack extension at failure of ${\left[90^{{}^{\circ}}/0^{{}^{\circ}}/\pm 45^{{}^{\circ}}\right]}_{3s}$ IM7-8552 notched laminates with varying hole diameters.

For a fixed width-to-diameter ratio ( $\frac{W}{2R}$ =6), the crack extension at failure *l* increases and tends to be stable with increasing hole size when hole diameter is larger than 0.5 mm as shown in Figure 3.

**Figure 4:**
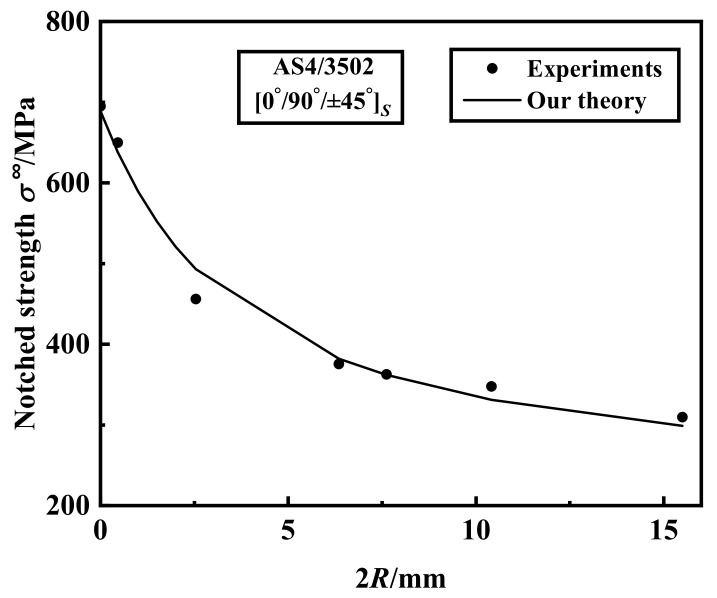
The uniaxial tensile strength of ${\left[0^{{}^{\circ}}/90^{{}^{\circ}}/\pm 45^{{}^{\circ}}\right]}_{s}$ AS4/3502 notched laminates with varying hole diameters.

The quasi-isotropic ${\left[0^{{}^{\circ}}/90^{{}^{\circ}}/\pm 45^{{}^{\circ}}\right]}_{s}$ notched laminates with AS4/3502 are chosen as the second batch of examples. All the laminates are with a constant plate width (*W*) equal to 152.4 mm, and with different hole diameters: 2*R* = 0.4572 mm, 2.54 mm, 6.35 mm, 7.62 mm, 10.414 mm, 15.494 mm. Figure 4 presents a comparison of the experimental results from Ref. [22] and the predictions of our theory for the uniaxial tensile strength in the *x*-direction. The figure shows that the predicted notched strength decreases with increasing hole diameter. The predicted results agree well with the experiments, and the maximum error is 8.1%.

**Figure 5:**
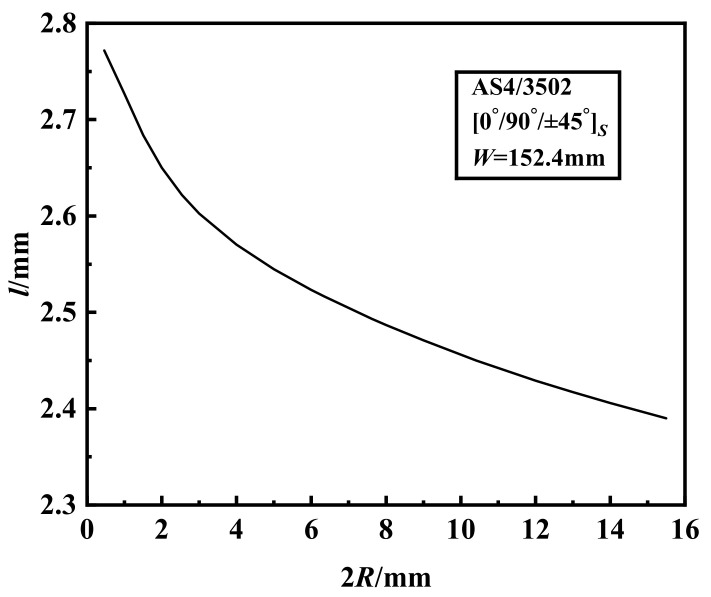
The crack extension at failure of ${\left[0^{{}^{\circ}}/90^{{}^{\circ}}/\pm 45^{{}^{\circ}}\right]}_{s}$ AS4/3502 notched laminates with varying hole diameters.

For a fixed plate width (*W* = 152.4 mm), the crack extension at failure *l* decreases with increase of the hole size as shown in Figure 5.

The symmetrical balanced laminates with material of Hexcel F593 epoxy system are chosen as the third batch of examples. All the laminates are with a constant plate width (*W*) and hole diameter (2*R*) equal to 32 mm and 6.35 mm, respectively. Table 2 presents a comparison of the experimental results from Ref. [30] and the predictions of our theory for the uniaxial tensile strength in the *x*-direction when $\frac{2R}{W}=0.2$ . The predicted results agree well with the experiments, and the maximum error is 10.7% when the laminate stiffness ratio of effective stiffness in the *x*-direction to *y*-direction is 1.55.

### 4.2. Importance Measurement Analysis of Material Properties to Notched Tensile Strength

In this section, we apply the importance measurement analysis of input variables in Section 3 to notched composite laminates. The statistical information for AS4/3501-6 of unidirectional laminas is listed in Table 3. It is assumed that the distribution type is log-normal, and the coefficient of variation for moduli and Poisson ratio is 0.1. The laminates are with a constant plate width (*W*) equal to 38.1 mm.

Firstly, the ${\left[45^{{}^{\circ}}/0^{{}^{\circ}}/-45^{{}^{\circ}}/90^{{}^{\circ}}\right]}_{2s}$ notched laminate under tension is taken as an example to analyse the influence of uncertain material properties on notched strength. The influence of hole diameters on importance measurement of material is also studied. The laminates are with different hole diameters: 2*R* = 2 mm, 3.81 mm, 6.35 mm. The total effect indices of material properties for the laminate with various hole diameters are shown in Table 4. It shows that longitudinal tensile strength *X_t_* has the greatest influence on the failure strength of the notched laminate with a fixed hole diameter, which means fiber rupture is the final damage mode. The notched strength is also affected by transverse modulus *E*_2_ and in-plane shear modulus *G*_12_. As to notched laminates with different hole diameters, the influence of material properties is almost unchanged with hole sizes. It means the influence of hole size on importance measurement of material is small.

Secondly, a notched laminate with one fixed hole diameter (2*R* = 3.81 mm) is chosen to analyse the influence of uncertain material properties on notched strength. The influence of stacking sequences on importance measurement of material is also studied. The laminates are with different stacking sequences: ${\left[-45^{{}^{\circ}}/90_{4}^{{}^{\circ}}/45^{{}^{\circ}}/90_{4}^{{}^{\circ}}/0^{{}^{\circ}}\right]}_{s}$ , ${\left[-45^{{}^{\circ}}/90_{2}^{{}^{\circ}}/45^{{}^{\circ}}/0^{{}^{\circ}}\right]}_{2s}$ , ${\left[45^{{}^{\circ}}/0^{{}^{\circ}}/-45^{{}^{\circ}}/90^{{}^{\circ}}\right]}_{2s}$ , ${\left[45^{{}^{\circ}}/0_{2}^{{}^{\circ}}/-45^{{}^{\circ}}/90^{{}^{\circ}}\right]}_{2s}$ . Table 5 shows total effect indices of material properties for the laminate with different stacking sequences. Results show that $X_{t}$ has the greatest influence on notched strength, and it increases with increasing stiffness ratio. However, the second important factor is different for notched laminates with different stacking sequences or stiffness ratios. For ${\left[-45^{{}^{\circ}}/90_{4}^{{}^{\circ}}/45^{{}^{\circ}}/90_{4}^{{}^{\circ}}/0^{{}^{\circ}}\right]}_{s}$ notched laminate, $G_{12}$ has the second largest influence on notched strength which means shear failure is dominant. $G_{12}$ decreases as stiffness ratio increases. For ${\left[-45^{{}^{\circ}}/90_{2}^{{}^{\circ}}/45^{{}^{\circ}}/0^{{}^{\circ}}\right]}_{2s}$ and ${\left[45^{{}^{\circ}}/0^{{}^{\circ}}/-45^{{}^{\circ}}/90^{{}^{\circ}}\right]}_{2s}$ notched laminates, $E_{2}$ has the second largest influence which means matrix failure is dominant, and it decreases with increasing stiffness ratio. However, $E_{1}$ has the second largest influence for ${\left[45^{{}^{\circ}}/0_{2}^{{}^{\circ}}/-45^{{}^{\circ}}/90^{{}^{\circ}}\right]}_{2s}$ notched laminate. The different influence of material properties means that the progressive failure processes are different for laminates with diverse stacking sequences.

**Figure 6:**
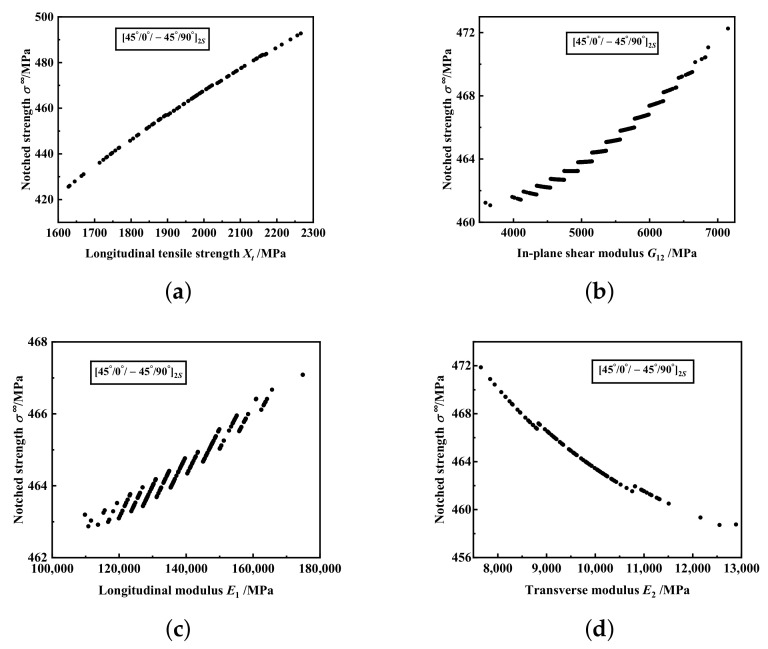
The influence of material properties on the tendency of failure strength for notched laminate with ${\left[45^{{}^{\circ}}/0^{{}^{\circ}}/-45^{{}^{\circ}}/90^{{}^{\circ}}\right]}_{2s}$ and 2*R* = 3.81 mm: (**a**) Longitudinal tensile strength *X_t_*. (**b**) In-plane shear modulus *G*_12_. (**c**) Longitudinal modulus *E*_1_. (**d**) Transverse modulus *E*_2_.

**Figure 7:**
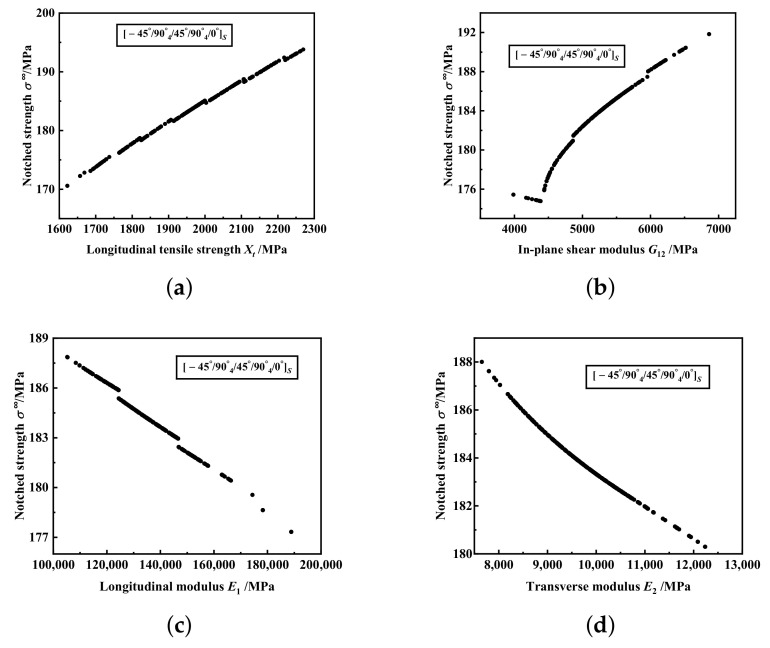
The influence of material properties on the tendency of failure strength for notched laminate with ${\left[-45^{{}^{\circ}}/90_{4}^{{}^{\circ}}/45^{{}^{\circ}}/90_{4}^{{}^{\circ}}/0^{{}^{\circ}}\right]}_{s}$ and 2*R* = 3.81 mm: (**a**) Longitudinal tensile strength *X_t_*. (**b**) In-plane shear modulus *G*_12_. (**c**) Longitudinal modulus *E*_1_. (**d**) Transverse modulus *E*_2_.

In order to analyse the effect of one input variable on tendency of response variable, it is assumed that one input variable is log-normal or normal distribution and other input variables are fixed to the corresponding mean values. Here, we assume that *X_t_*, *G*_12_, *E*_1_ and *E_2_* are log-normal, and the influence of theses four material properties on the tendency of failure strength for ${\left[45^{{}^{\circ}}/0^{{}^{\circ}}/-45^{{}^{\circ}}/90^{{}^{\circ}}\right]}_{2s}$ and ${\left[-45^{{}^{\circ}}/90_{4}^{{}^{\circ}}/45^{{}^{\circ}}/90_{4}^{{}^{\circ}}/0^{{}^{\circ}}\right]}_{s}$ notched laminates with 2R=3.81 mm are shown in Figure 6 and Figure 7, respectively. Both two figures show that the notched strength increases with increasing *X_t_* and *G*_12_, but with decreasing *E*_2_. With increasing *E*_1_, the notched strength of ${\left[45^{{}^{\circ}}/0^{{}^{\circ}}/-45^{{}^{\circ}}/90^{{}^{\circ}}\right]}_{2s}$ laminate increases, but that of ${\left[-45^{{}^{\circ}}/90_{4}^{{}^{\circ}}/45^{{}^{\circ}}/90_{4}^{{}^{\circ}}/0^{{}^{\circ}}\right]}_{s}$ notched laminates decreases.

## 5. Conclusions

In summary, a developed failure theory and importance measurement of the material properties are discussed in this paper. Firstly, a failure theory for fiber-reinforced composite laminates with a circular hole is developed, by combining finite fracture mechanics criterion with improved Puck’s failure theory. In this theory, only basic material properties of unidirectional laminas are needed to predict notched strength of the laminate, without in advance mechanical information of the laminate. The predicted results are also in good agreement with experimental results. Secondly, importance measurement of the uncertain material properties is analysed. Results show that longitudinal tensile strength has the greatest influence on the notched strength, and increases with increasing stiffness ratio. The second important factor is different for the notched laminate with different stacking sequences. In-plane shear modulus has the second largest influence for notched laminate with stiffness ratio 0.22, and decreases as stiffness ratio increases. Longitudinal and transverse modulus are also important to notched strength. The notched strength increases with increasing longitudinal tensile strength and in-plane shear modulus, but with decreasing transverse modulus.

## Figures and Tables

**Table 1:** Material properties.

Material	IM7-8552	AS4/3502	Hexcel F593epoxy System
Longitudinal modulus $E_{1}\;\left(\mathrm{GPa}\right)$	171.4 [5]	140 [22]	120 [30]
Transverse modulus $E_{2}\;\left(\mathrm{GPa}\right)$	9.1 [5]	10.3 [22]	9 [30]
In-plane shear modulus $G_{12}\;\left(\mathrm{GPa}\right)$	5.3 [5]	5.7 [22]	4.7 [30]
In-plane Poisson ratio $\nu_{12}$	0.3 [5]	0.3 [22]	0.35 [30]
Longitudinal tensile strength $X_{t}\;\left(\mathrm{MPa}\right)$	2326 [5]	1862 [22]	1451 [30]
Longitudinal compressive strength $X_{c}\;\left(\mathrm{MPa}\right)$	1200 [5]	1483 [22]	1306 [30]
Transverse tensile strength $Y_{t}\;\left(\mathrm{MPa}\right)$	62 [5]	51.7 [22]	98 [30]
Transverse compressive strength $Y_{c}\;\left(\mathrm{MPa}\right)$	199.9 [5]	206.9 [22]	215 [30]
In-plane shear strength $S_{12}\;\left(\mathrm{MPa}\right)$	92.3 [5]	81 [22]	40 [30]
Fracture toughness of the $0^{{}^{\circ}}$ plies $K_{\mathrm{IC}}^{0}\;\left(\mathrm{MPa}\sqrt{m}\right)$	85.2 [26]	80.2 ^1^	157.4 ^2^

^1^ Derived from fracture toughness of [0°/90°/±45°]_*s*_ laminate [22] and Equations (14)–(18). ^2^ Derived from notched strength from tests of [45°/ $0_{2}^{{}^{\circ}}$ /−45°/ $0_{2}^{{}^{\circ}}$ /45°/ $0_{2}^{{}^{\circ}}$ /−45°/90°]_*s*_ laminate [30] and Equations (14)–(18)

**Table 2:** The notched tensile strength of symmetrical balanced laminates with material of Hexcel F593 epoxy system.

Label	Stacking Sequence	Stiffness Ratio (Exp.)	$\sigma^{\infty }$ (MPa) (Exp.)	$\sigma^{\infty }$ (MPa) (Pre.)	Error
1	${\left[-45^{{}^{\circ}}/90_{4}^{{}^{\circ}}/45^{{}^{\circ}}/90_{4}^{{}^{\circ}}/0^{{}^{\circ}}\right]}_{s}$	0.22	211	199	−5.7%
2	$\left[-45^{{}^{\circ}}/90_{2}^{{}^{\circ}}/45^{{}^{\circ}}/90_{2}^{{}^{\circ}}/\right.$ ${\left.\;-45^{{}^{\circ}}/90_{2}^{{}^{\circ}}/45^{{}^{\circ}}/0^{{}^{\circ}}\right]}_{s}$	0.35	244	243	−0.4%
3	${\left[-45^{{}^{\circ}}/90_{2}^{{}^{\circ}}/45^{{}^{\circ}}/0^{{}^{\circ}}\right]}_{2s}$	0.65	337	355	5.3%
4	${\left[45^{{}^{\circ}}/0^{{}^{\circ}}/-45^{{}^{\circ}}/90^{{}^{\circ}}\right]}_{3s}$	1.00	378	417	10.3%
5	${\left[45^{{}^{\circ}}/0_{2}^{{}^{\circ}}/-45^{{}^{\circ}}/90^{{}^{\circ}}\right]}_{2s}$	1.55	486	538	10.7%

**Table 3:** Statistical information for AS4/3501-6.

Random Variables	Mean Value	Standard Deviation	Distribution Type
Longitudinal modulus $E_{1}\;\left(\mathrm{GPa}\right)$	138 [26]	13.8	Log-Normal
Transverse modulus $E_{2}\;\left(\mathrm{GPa}\right)$	9.65 [26]	0.965	Log-Normal
In-plane shear modulus $G_{12}\;\left(\mathrm{GPa}\right)$	5.24 [26]	0.524	Log-Normal
In-plane Poisson ratio $\nu_{12}$	0.3 [26]	0.03	Log-Normal
Longitudinal tensile strength $X_{t}\;\left(\mathrm{MPa}\right)$	1969 [19]	196.9 [19]	Log-Normal
Longitudinal compressive strength $X_{c}\;\left(\mathrm{MPa}\right)$	1480 [19]	177.6 [19]	Log-Normal
Transverse tensile strength $Y_{t}\;\left(\mathrm{MPa}\right)$	48 [19]	2.88 [19]	Log-Normal
Transverse compressive strength $Y_{c}\;\left(\mathrm{MPa}\right)$	200 [19]	16 [19]	Log-Normal
In-plane shear strength $S_{12}\;\left(\mathrm{MPa}\right)$	79 [19]	8.69 [19]	Log-Normal
Fracture toughness of the $0^{{}^{\circ}}$ plies $K_{\mathrm{IC}}^{0}\;\left(\mathrm{MPa}\sqrt{m}\right)$	82.8 [26]	-	-

**Table 4:** Importance factors regarding material properties for ${\left[45^{{}^{\circ}}/0^{{}^{\circ}}/-45^{{}^{\circ}}/90^{{}^{\circ}}\right]}_{2s}$ notched laminate (%).

Random Variables	2R=2 mm	2R=3.81 mm	2R=6.35 mm
Longitudinal modulus $E_{1}$	0.11	0.36	0.33
Transverse modulus $E_{2}$	0.72	1.74	1.77
In-plane shear modulus $G_{12}$	0.64	0.73	0.73
In-plane Poisson ratio $\nu_{12}$	0.01	0.01	0.01
Longitudinal tensile strength $X_{t}$	98.65	96.59	96.85
Longitudinal compressive strength $X_{c}$	0	0	0
Transverse tensile strength $Y_{t}$	0.06	0.07	0.07
Transverse compressive strength $Y_{c}$	0	0	0
In-plane shear strength $S_{12}$	0.04	0	0.05

**Table 5:** Importance factors regarding material properties for notched laminate with 2R=3.81 mm (%).

Random Variables	${\left[-45^{{}^{\circ}}/90_{4}^{{}^{\circ}}/45^{{}^{\circ}}/90_{4}^{{}^{\circ}}/0^{{}^{\circ}}\right]}_{s}$ (Stiffness Ratio 0.22)	${\left[-45^{{}^{\circ}}/90_{2}^{{}^{\circ}}/45^{{}^{\circ}}/0^{{}^{\circ}}\right]}_{2s}$ (Stiffness Ratio 0.65)	${\left[45^{{}^{\circ}}/0^{{}^{\circ}}/-45^{{}^{\circ}}/90^{{}^{\circ}}\right]}_{2s}$ (Stiffness Ratio 1)	${\left[45^{{}^{\circ}}/0_{2}^{{}^{\circ}}/-45^{{}^{\circ}}/90^{{}^{\circ}}\right]}_{2s}$ (Stiffness Ratio 1.55)
Longitudinal modulus $E_{1}$	4.06	0.19	0.36	1.48
Transverse modulus $E_{2}$	3.85	3.05	1.74	0.79
In-plane shear modulus $G_{12}$	15.08	2.29	0.73	0.14
In-plane Poisson ratio $\nu_{12}$	0.01	0.01	0.01	0.02
Longitudinal tensile strength $X_{t}$	68.2	92.59	96.59	99
Longitudinal compressive strength $X_{c}$	0	0	0	0
Transverse tensile strength $Y_{t}$	0.67	0.11	0.07	0.03
Transverse compressive strength $Y_{c}$	0	0	0	0
In-plane shear strength $S_{12}$	0.06	0.04	0	0.02

## Data Availability

Not applicable.
